# Protective Effect of Hyperbaric Oxygen Treatment on Axon Degeneration after Acute Motor Axonal Neuropathy

**DOI:** 10.1155/2021/6627779

**Published:** 2021-11-08

**Authors:** Ni Komang Sri Dewi Untari, Kurnia Kusumastuti, Guritno Suryokusumo, I Ketut Sudiana

**Affiliations:** ^1^Department of Hyperbaric, Drs. Med. Rijadi S. Phys. Naval Health Institute, Surabaya, Indonesia; ^2^Department of Neurology, Faculty of Medicine, Hang Tuah University, Surabaya, Indonesia; ^3^Department of Neurology, Dr. Ramelan Navy Hospital, Surabaya, Indonesia; ^4^Hyperbaric Medicine, Basic Medical Science, Airlangga University, Surabaya, Indonesia; ^5^Department of Neurology, Faculty of Medicine, Airlangga University, Surabaya, Indonesia; ^6^Department of Hyperbaric, Faculty of Medicine, Pembangunan Nasional University, Jakarta, Indonesia; ^7^Department of Pathology Anatomy, Faculty of Medicine, Airlangga University, Surabaya, Indonesia

## Abstract

**Objectives:**

Acute motor axonal neuropathy (AMAN) is a disease that leads to acute flaccid paralysis and may result from the binding of antibody and antigen to the spinal cord. The objective of this study is to evaluate the protective effect of hyperbaric oxygen treatment (HBOT) on axon degeneration of the spinal cord and sciatic nerve of the AMAN model rabbit. Axonal degeneration was assessed by evaluating glutathione (GSH) activity, interleukin-1*β* (IL-1*β*) expression, and clinical and histopathological features.

**Methods:**

Twenty-one New Zealand rabbits were divided into three groups. The treatment group was exposed to 100% oxygen at 2.4 ATA 90 minutes for 10 days at a decompression rate of 2.9 pounds per square inch/minute. GSH level was evaluated using an enzyme-linked immune-sorbent assay. An expression of IL-1*β* in the spinal cord was determined by immunohistochemistry. Clinical appearances were done by motor scale and body weight. Histological features observed neuronal swelling and inflammatory infiltration in the sagittal lumbar region and the undulation of the longitudinal sciatic nerve.

**Results:**

Rabbits exposed to HBO had high GSH activity levels (*p* < 0.05) but unexpectedly had high IL1*β* expression (*p* > 0.05). In addition, the HBO-exposed rabbits had a better degree of undulation, the size of neuronal swelling was smaller, the number of macrophages was higher, and motor function was better than the AMAN model rabbits (*p* < 0.05).

**Conclusions:**

These findings indicate that HBO therapy can decrease axon degeneration by triggering GSH activity, increasing IL-1*β* level, and restoring tissues and motor status. In conclusion, HBO has a protective effect on axon degeneration of the spinal cord and sciatic nerve of the AMAN model rabbit.

## 1. Introduction

Globally, Guillain–Barré Syndrome (GBS) represents the most common disease, which results in acute flaccid paralysis. Histopathological features divide GBS into demyelinating and axonal subtypes, which are called acute inflammatory demyelinating polyneuropathy (AIDP) and acute motor axonal neuropathy (AMAN). The existing evidence suggests that peripheral nerve-binding autoantibodies followed by complement deposition may result in neuronal damage in both AIDP and AMAN [1]. AMAN constitutes a form of GBS, which is commonly found in China, Japan, and Central and South America with a frequency of around 35–65%. Patients with AMAN are associated with motor neuron damage in the nodes of Ranvier [2]. AMAN is generally associated with *Campylobacter jejuni* (*C. jejuni*) and *Zika virus* infection [3]. Disability is often found in patients treated with standard therapy for months [4]. On the other hand, conventional therapy is unsatisfactory due to its high cost and some complications.

Hyperbaric oxygen therapy (HBOT) for patients with stroke, atherosclerosis, cerebral palsy, intracranial hypertension, headache, brain injury, and bone marrow injury seems promising but requires further observations [5]. This therapy involves inhalation of pure oxygen in a high-pressure air room 1–3 times more than normal atmospheric pressure. HBO increases the proliferation and differentiation of endogenous germ cells. It also increases arterial oxygen partial pressure, blood oxygen levels in the central nervous system, and aerobic metabolism in the central nervous system and reduces secondary damage in the bone marrow injury [6].

The HBO therapy increases GSH indicating a very efficient antioxidant mechanism to protect against oxidative stress [7]. GSH represents an essential cellular redox buffer and plays an important role in the detoxification of not only MDA but also NO and other ROS-induced fat peroxidation products, such as 4-hydroxyinonenal (4-HNE) [8]. IL-1*β* is an interleukin-1 cytokine, which is produced by activated macrophages as a proprotein, which is proteolytically processed to its active form by caspase [9].

The main obstacle to AMAN therapy is the disability which may last for months. This is because the inflammatory process and molecular mimicry are still ongoing. Clinical observations show that HBO accelerates motor recovery in AMAN patients. These observations raise the possibility that HBO can decrease axon degeneration. In this study, we examine GSH activity, IL-1*β* level, histopathology of the spinal cord, and sciatic nerve and motor status in the AMAN model.

## 2. Methods

### 2.1. Research Design

This study was conducted in a laboratory and was a true experiment because all subjects were randomly selected, involving a control group and confounding variable, which could be controlled. The study used a “randomized control group posttest design.” The sampling technique used here was a simple random sampling method.

### 2.2. Inducing Acute Motor Axonal Neuropathy Model

Male New Zealand rabbits (age 2–3 months, weight 1.8–2.2 kg) were obtained from the Firamichi Rabbit Farm. On arrival, all rabbits had clear eyes, shiny fur, and agile movement, indicating that the rabbits were in healthy condition. Adaptation took place for 14 days at Naval Health Center, Surabaya, Indonesia. The twenty-one New Zealand rabbits were divided into control (C), AMAN (A), and AMAN + HBO (AH) groups (*n* = 7 in each group). There was no significant difference in baseline characteristics, such as Tarlov score and body weight (*α* > 0.005) at the onset. The experiments were conducted under the guidelines for the care and use of laboratory animals from the Naval Health Institute. Rabbits did not experience unnecessary suffering.

All rabbits were housed in normobaric conditions, namely, 1013 hPa with 20.9% oxygen. All rabbits were fed ad libitum. The room was maintained in a controlled 12-hour light/dark cycle (dark period from 20.00 to 08.00) at 22 ± 2°C with a relative humidity of 45–55%.

Yuki's method was used to create a AMAN model. They were injected subcutaneously to the back with 2.5 mg of total ganglioside from pig brain, 1 mg of keyhole limpet hemocyanin, and 0.5 ml of complete Freund's adjuvant at 3-week intervals until disease onset or maximal sensitization (five times). The AMAN model animals were obtained after 10 weeks.

### 2.3. Hyperbaric Oxygen Therapy and Animal Groups

Animals were randomly assigned to one of the following three groups. The control group did not receive ganglioside injections and did not follow HBO. In the disease model group, all subjects developed AMAN and were exposed to normobaric air (21% oxygen at 1 ATA). The HBO treatment group, after the subjects developed AMAN, were all treated with HBO (100% oxygen at 2.4 ATA) for 90 minutes at a decompression rate of 2.9 pounds per square inch/minute.

### 2.4. Assessing Hindlimb Locomotor Function

The clinical scores of rabbits were blinded to observers and monitored weekly. Disease onset was defined as a clinical score of 3 or less. The motor test was done to measure the functional recovery of the hind limbs of rabbits according to the procedure described by the Tarlov score where 0 indicates no hind limb activity, unable to bear body weight; 1, an activity of the hind limbs but unable to bear the body weight; 2, a frequent or vigorous activity of the hind limbs but unable to bear the body weight; 3, the hind limbs can bear the body weight and the animal can take 1-2 steps; 4, animals can walk with mild interference; and 5, animals can run normally [6].

### 2.5. Assessing Axon Degeneration of the Spinal Cord

Immediately after the assessment of motor performance on week 18 after AMAN, the animals were terminated for IHC staining. All rabbits in each group were anesthetized using 10% chloral hydrate (350 ml/kg) and fixed with paraformaldehyde. Neuropathic tissues were removed and dehydrated with an ethanol gradient. There are two types of tissues. The lumbar side was cut sagittally and the sciatic nerve was cut longitudinally to a thickness of 20 m using a cryostat. These sections were stained with hematoxylin for 5 minutes, rinsed with tap water, differentiated using hydrochloric acid for 10 seconds, rinsed with tap water for 10 minutes, stained with eosin for 7 minutes, dehydrated through an ethanol gradient, made transparent with xylene, and then mounted.

### 2.6. IL-1*β* Expression in the AMAN Lumbal Spinal Cord and Ischiadicus Nerve as Detected by Immunohistochemistry

For the determination of IL-1*β* in the AMAN model, samples were taken after the HBO, or in the equivalent time for the AMAN model rabbits. Alternative sections of tissue removed for HE staining were used for immunohistochemistry. Expression of IL-1*β* in animal models of the lumbar spinal cord was determined using rabbit monoclonal antibody anti-IL-1*β* (Santa Cruz Biotechnology, Inc., Santa Cruz, CA) by immunohistochemical technique. The staining method used the avidin-biotin complex.

### 2.7. Glutathione Value in the AMAN Lumbal Spinal Cord and Ischiadicus Nerve as Analyzed in ELISA

For the determination of GSH in the AMAN model, samples were taken after HBO, or the equivalent time for AMAN model rabbits. GSH levels in plasma were measured using GSH antibodies (E1067Rb, Bioassay Technology Laboratory) with an enzyme-linked immunosorbent assay (ELISA) reader (Zenix-320 microplate reader). The captured GSH levels were determined by measuring the absorbance at a wavelength of 450 nm using a spectrophotometer.

### 2.8. Statistical Analysis

Tarlov baseline scores, axon degeneration scores, GSH concentrations, and the number of positive IL-1*β* cells from the three groups were compared by an analysis of variance (ANOVA). This was done to examine the interaction of treatment with time and the effect of treatment from time to time. Data were presented as mean ± standard deviation (SD), and statistical significance was set at *p* < 0.05. IBM SPSS statistics was used to analyze all data.

## 3. Results

### 3.1. Tarlov Scale

Fourteen rabbits injected with IgGM1 show flaccid paralysis of locomotor hind; the first time appeared ranging from 75 to 110 days (mean 95 days) after the first inoculation. In contrast, none of the 7 control rabbits show weakness locomotor until the end of examination (17 weeks).

Locomotor weakness becomes progressive 4–13 days (median 5 days) after the start of the weakness in 14 rabbits until reaching a constant value. One rabbit from the AMAN group and two from the AMAN-HBO group had tremor several days before the onset of locomotor weakness. Tetraparese occurred rapidly in one rabbit of the AMAN group with progressive gasping and died at week 16.

The Tarlov scale was examined every week, suggesting that on week 17, the right time for HBO therapy was immediately implemented, showing that the Tarlov scale was significantly different between the control group and the AMAN group (*p*=0.006) (Table 1).

**Figure 1:**
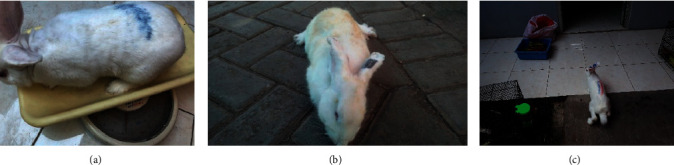
Clinical picture of control, AMAN, and AMAN rabbits treated by HBO. (a) Median Tarlov score of “5” at control group. (b) Median Tarlov score of “3” at AMAN group. (c) Median Tarlov score of “4” at AMAN with HBO group.

Figure 1 shows that the AMAN-induced hind limb motor deficits remarkably improved with HBO therapy on day 10 after HBO. Rabbits can walk with mild impairment.

### 3.2. Weight

The baseline weights of the rabbits were homogeneous (*α* = 0.864). Fourteen rabbits immunized with GM1 began to lose weight in approximately nine weeks. When a rabbit started losing weight consistently for two weeks, it is considered as the onset of the disease. After experiencing weight loss, they began experiencing locomotor weakness. Weight in this group when its decrease was stopped by 0.1 to 0.3 was calculated from the onset of clinical symptoms (weeks 8 to 10).

There was no weight loss in the control group. In contrast, there was an increase in body weight in the range of 0.7 to 1 kg in AMAN + HBO groups. Body weight was significantly different between the control and AMAN + HBO groups (*p*=0.001) (Table 2).

### 3.3. Hematoxylin–Eosin Staining

The median of inflammatory infiltration was 1 in the control group.  Table 3 shows that the median score of inflammatory infiltration in the AMAN group was 3. Based on the Kruskal–Wallis test in the two groups injected with GM1, there was a significant difference between the AMAN and AMAN + HBO groups (*p* < 0.05).

**Figure 2:**
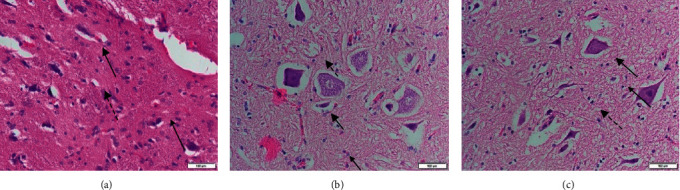
Hematoxylin and eosin-stained histopathological images at the cross section of the lumbar spinal cord. Microscopic views under 200× magnification show normal white matter at the control group (a), moderate axonal degeneration at the AMAN group (b), and mild axonal degeneration at AMAN with the HBO group (c). (Bold line arrows indicate neuronal edema. Thin line arrows indicate macrophage infiltration. Dotted line arrows indicate lymphocytic infiltration.) The scale bars represent 100 *μ*m.

Figure 2 shows a cross section of the control rabbit lumbar spinal cord (C) showing normal-sized neuron cells without intraneural edema. On the other hand, neural edema was found in the AMAN group (A and AH). The average number of macrophages was below 10 and the number of lymphocytes below 3 in preparation C. Meanwhile, for the GM1 injection, the average number of inflammatory infiltrates ranged from six to twenty.

There were differences in histopathological signs of inflammatory infiltration in the lumbar spinal cord of the guinea pigs. The AMAN group showed a more severe inflammatory process compared with AMAN + HBO. There was no inflammatory process in the control group.

**Figure 3:**
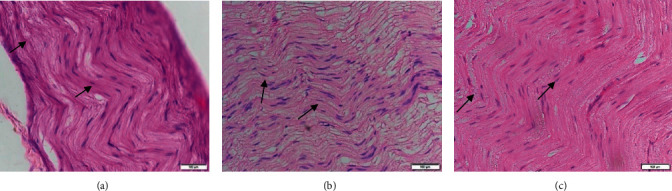
Characteristic of the ischiadicus nerve's undulation with HE staining. Microscopic views under 200x magnification show normal white matter at the control group (a), moderate axonal degeneration at the AMAN group (b), and mild axonal degeneration at AMAN with the HBO group (c). The bold line arrows indicate the undulation height. Thin line arrows indicate Schwan cells. The scale bars represent 100 *μ*m.

In the HE-stained lumbar fragment, tissue patterns can be observed, which makes it possible to recognize gray matter and white matter. The longitudinal section showed a unique undulation pattern with a straight line height between the two mountain-like formations of more than 3 cm. There were no signs of axonal degeneration in the normal rabbit sciatic nerve.  Figure 3 indicates the longitudinal section of the normal rabbit sciatic nerve with characteristic undulations in the control group. On the other hand, the undulation pattern started to decrease in the two GM1-injected groups (AMAN and AMAN + HBO).

In the AMAN rabbit sciatic nerve, a characteristic undulation tissue pattern was observed from the peripheral nerves with Wallerian degeneration. There was structural disorganization with a straight line height between the two mountain-like formations of less than 2.5 cm (Figure 3). In some rabbits, this undulation almost completely disappeared. There was a significant difference between axon degeneration scores at week 18 (*p*=0.01). In the ischiadicus nerve of AMAN + HBO, the histochemical reaction to the collagen fibers was intense and the fibers tended to orient in place.

### 3.4. Antioxidant Activity

Descriptive data on GSH levels of the model rabbits are presented in Table 4. Table 4 shows the highest mean GSH levels in the control group (189091,71) and the lowest GSH levels in the AMAN group (70382,57).

**Figure 4:**
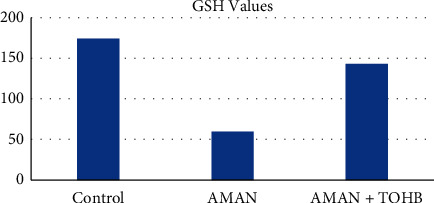
GSH analysis with ELISA method.

To explain whether GSH can be induced by HBO, an analysis of GSH activity in blood was carried out, revealing that GSH after HBO therapy showed more GSH activities (Figure 4) compared with the AMAN (*p* < 0.05).

### 3.5. Proinflammatory Cytokines

Table 5 describes an expression of IL-1*β* in the rabbit model. The highest mean IL-1 expression was seen in the AMAN + HBO group (4,714) and the lowest in the control group (3.539).

**Figure 5:**
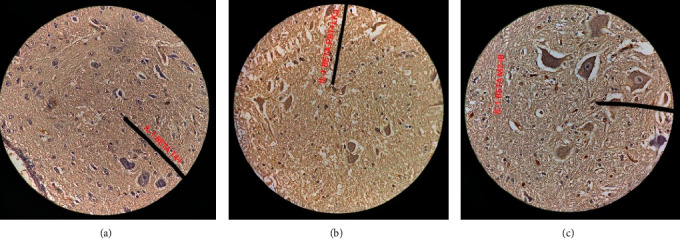
The expression of IL-1*β* with an immunohistochemistry technique. Microscopic views under 200x magnification show IL-1*β* expression in microglia cells in the control group (a), IL-1*β* expression in microglia cells in the AMAN group (b), and IL-1*β* expression in microglia cells in the AMAN with HBO group (c). (Black arrows show colored microglia cells brown which gives a positive reaction to anti-IL-1*β* monoclonal antibody in the lumbar vertebral bone marrow.)

To elucidate whether proinflammatory cytokine (IL-1*β*) production is affected by HBO in AMAN, IHC staining was conducted 1 h after discontinuation. It was found that compared with AMAN rabbits, the AMAN + HBO rabbits paradoxically had higher IL-1*β* production (Figure 5).

## 4. Discussions

Acute motor axonal neuropathy, as a type of axonal GBS, constitutes an acute onset and immune-mediated disorder of the peripheral nervous system [10]. Epidemiologically, AMAN is associated with previous *C. jejuni* infection. Autoantibodies against the gangliosides GM1 and GD1a are found in 40% and 30% of patients, respectively. Autopsy studies of AMAN patients and pathology experimental models of AMAN showed varying degrees of Wallerian-like degeneration with IgG and complement deposits in nodes of Ranvier with no or little demyelination or lymphocytic inflammation [11].

Some patients recovered during the first year, especially the first 6 months. However, a large part of patients continue to recover well until the second year and often beyond [10]. Estimated costs in the United States are $110,000 for health care while hospitalized and $360,000 for lost productivity per patient [12]. Plasma exchange and intravenous immunoglobulin are effective immune therapies for pediatric and adult patients when administered during the first few weeks. Together with supportive care, the two standard therapies can minimize the risk of death and improve clinical appearances [13]. Immune therapy does not reduce mortality in GBS. Causes of death are usually related to the disease or secondary complications that occur in the hospital due to the long course of the disease [14].

All rabbits immunized with GM1 showed signs and symptoms of weakness very similar to those with axonal type GBS. Nagai observed that rabbits, which were intensively immunized with bovine brain gangliosides or certain molecules of ganglioside species, such as GD1a and GM1 in CFA would undergo various neurological symptoms and signs particularly similar to experimental allergic encephalomyelitis [15]. In this study, some rabbits experienced early symptoms such as tremors. On average at week 8, some rabbits began to experience a reduction in intake. Two weeks later, weight loss was recorded from 0.1 to 0.2 kg. Clinical symptoms of motor weakness appear a week after with a decrease in the agility of back motion until it can only move the front. One rabbit died on day 40 after symptoms begin.

*C. jejuni* infection, as a cause of bacterial gastroenteritis, is the leading cause of AMAN worldwide. The related serotypes are HS: 19 and HS: 41 [16]. The disease may persist for more than six weeks after onset. Approximately, 20%–30% of patients developed complications, such as respiratory failure requiring mechanical ventilation [17]. Kuwabara and Yuki said that a loss of sodium channel cluster nodes and paranodal myelin release was found in the AMAN rabbits. Antibodies against gangliosides and complement will directly inactivate sodium channels [18].

As the simplest description, HE is the most frequently used stain in peripheral nerves by light microscopy [19]. Although this staining is not an ideal method to analyze the process of nerve regeneration, its electropolar nature will generate pink and purple colors with varying intensities in the neural elements, so that each degenerative nerve will be displayed as a vacuole (vacuole focus). The empty zone is a characteristic of myelin with this staining [20]. The sciatic nerve shows mild-to-moderate Wallerian-like degeneration, with progressively flattened undulations. Macrophage invasion was found in the perivascular endoneurial area, but there was no lymphocyte infiltration in any of the sciatic nerve regions. These results indicate that this syndrome may be induced by an autoimmune response exerted by ganglioside-responding cells. Yuki et al. who investigated the characterization of chronic axonal degeneration in bone marrow injury stated that there were no histopathological criteria. They only divided the severity by looking at axon loss, demyelination, and vacuolation [21].

The main exposure of GM-1 ganglioside against the spinal cord caused the death of several neurons. However, neurons died after eight weeks. The local inflammatory response in the AMAN spinal cord is believed to significantly contribute to the evolution of secondary events. There is evidence that early inflammatory events triggered tissue damage. The inflammatory response, characterized by neutrophil infiltration and microglia activation, develops within hours, which is followed by the expression of proinflammatory cytokines, including IL-1*β* [9].

Axon degeneration begins with an irreversible hypoxic process. Reduction oxidative stress may be associated with an overproduction of lipid molecules and/or a significant reduction in the effectiveness of antioxidant defenses. Inadequate antioxidant cellular mechanisms may also be involved in nerve damage [11]. The activities of cellular antioxidants such as superoxide dismutase (SOD) and GSH may be important for this process. GSH-px catalyzes the reduction of hydrogen peroxide by two glutathione molecules that serve as a part of the reactive oxygen species defense system [22]. In cases of early-stage bone marrow injury, impaired microcirculation may trigger localized hypoxia and ischemia and lead to immune inflammation. This also happens in case of the AMAN. During this period, TNF-*α*, IL-1, and other inflammatory factors are released to make cytotoxicity and formation of scar tissue. The ongoing occurrences of hypoxia, bone marrow ischemia, and metabolic abnormalities are conditions that are not favorable to axonal and neuronal regeneration, resulting in secondary injury [6].

Hyperbaric oxygen treatment (HBOT) is defined by the Undersea and Hyperbaric Medical Society (UHMS) as a treatment in which the patient periodically inhales 100% oxygen in a hyperbaric chamber with pressure in excess of the surface pressure [23]. Hyperbaric oxygen can add up tissue oxygenation and stimulate the formation of H_2_O_2_ as a secondary messenger for phosphorylation of NF-ҡB. This transcriptional protein plays an important and quick role in various genes in response to extracellular stimuli. Many aspects of the inflammatory response are also regulated by NF-kB, including the production of the complement cascade and the induction of proinflammatory cytokines such as IL1 and TNF-*α* [24].

With significantly higher levels of GSH and CAT enzymes in the HBO group compared with the myocardial infarction and control group, Oliveira et al. concluded that HBO can reduce mortality by enhancing redox control in rat hearts in the acute myocardial infarction. Ilhan et al. found that GSH-PX and CAT levels were significantly higher in the control group and negative treatment groups, while concentrations of MDA, NSE, S100*β*, and NO were lower in bone marrow injury [25].

It has been shown that after experimental SCI, TNF-*α* and IL-1*β* levels are significantly increased in the injured spinal cord in the first few hours after injury. The existing evidence also said that TNF-*α* and IL-1*β* play a considerable role in the induction of inducible nitric oxide synthase (iNOS), cyclooxygenase-2 (COX-2), and reactive oxygen species (ROS), which are responsible for the secondary neuronal damage encountered after the SCI. The studies by Tai et al. showed that HBO may increase bone marrow inflammation after bone marrow injury by reducing TNF-*α* and IL-1*β* production, but increasing IL-10 production [26]. In the case of irradiated laryngeal tissue, HBO can decrease TNF-*α*, IL-1*β* levels, and tissue inflammation and increase IL-10 levels [27].

The following is the mechanism of action of HBO therapy in improving the microenvironment of the injured bone marrow. Bone marrow injury may result in local edema, hypoxia, and hemorrhage. HBO therapy reduces hypoxia-induced neuronal apoptosis by increasing blood oxygen concentration in the injured bone marrow and oxygen diffusion capacity [28]. HBO regulates AQP4/9 protein and gene expression by reducing inflammation in the injured bone marrow. HBO therapy also corrects acidosis in injured bone marrow, improves microcirculation, plays a role in the defense of energy metabolism, and promotes recovery of nerve function after reversible injury [6].

A large number of studies in various cases revealed that HBOT decreased inflammatory agents, including IL-1*β*. In cases of temporomandibular joint osteoarthritis, HBOT protects chondrocytes against IL-1*β*-induced apoptosis through the PI3K/AKT30 marker pathway [29]. Bosco et al. stated that there is a decrease in plasma levels of TNF-*α*, IL-6, and IL-1*β* in avascular femoral head necrosis (AVNFH) which reduces bone marrow eczema and pain complaints [30]. THBO is also said to enhance the inflammatory response in brain tumor patients by reducing TNF-*α* and IL-6 levels, reducing mean cerebral artery flow, and effectively reducing cerebral artery spasm [31].

Ozden et al. conducted a study on mice treated with HBO for 7 days by observing rat liver tissues. Hyperbaric oxygen can increase antioxidant enzymes (MDA, GSH, Cu, ZnSOD) as well as Cu and Zn ions which are metal ions that form antioxidant SOD [32]. HBO can increase the formation of antioxidant enzymes such as Mn, Cu/Zn superoxide dismutase, glutathione peroxidase, and catalase. When the main nonenzymatic antioxidant system improves, the glutathione/cysteine system will improve too. The protective effect arose in the first hour after exposure and was still found 24–72 hours after the last HBO therapy [33].

In the study, AMAN in rabbits resulted in moderate axonal degeneration characterized by neuronal swelling, macrophage infiltration, decreased antioxidant (GSH), and proinflammatory cytokine (IL-1*β*) production, leading to tissue damage and hind limb locomotor dysfunction. Our hope was that treating AMAN rabbits with HBO therapy significantly reduced the level of spinal cord inflammation, macrophage infiltration, proinflammatory cytokine production; increased antioxidant activity; and improved hind limb locomotor dysfunction.

Indeed, there is a contradiction, namely, an increase in proinflammatory IL-1*β*. This may happen because of three things. First, it may be due to an increase after the decline on day 5. This study clearly demonstrates that treatment with HBO reduces the progression of AMAN-associated inflammation in the first stage and associated tissue injury. Maybe there is an increase again after that stage. In a different study, Susilo et al. concluded that HBO 2.4 ATA for 3 × 30 minutes could increase 5 times the expression of e-NOS, TNF-*α*, VEGF, and wound repair, but there was no wound repair 10 times [24].

Second, there is a high increase from two sources. The first is from inflammation due to AMAN and second from continuous production of ROS that cannot be tolerated by increased GSH. The aerobic metabolic pathway resulting in the accumulation of succinate in activated M1 macrophages stabilizes hypoxia-inducible factor (HIF-1a), thereby inducing IL-1*β*. In addition, ROS activate inflammatory caspase-1. ROS induce a powerful antioxidant N-acetylcysteine, which facilitates IL-1 secretion [34].

Third, it is possible that most of the HBOT-stimulated GSH does not pass through the IL-1*β* pathway. GSH activation may happen via another interleukin, possibly IL-6, IL-10, or others. Various studies on bone marrow injury did show a decrease in IL-1*β* after HBO therapy. The main difference between bone marrow injury and GBS is the time of occurrence and the type of disease. It is possible that the acute state and trauma type are more significant for IL-1*β* than the subacute and autoimmune states. Avtan et al. even concluded that HBOT did not exert a maximal protective effect on the integrity of the blood-brain barrier under septic conditions and even led to disruption of the blood-nerve barrier in intact animals [35].

Finally, it should be emphasized that, in this study, the fact that HBO is administered after AMAN requires further research. The long-term efficacy, reliability of this therapy, and minimal complications make OHB therapy a promising new therapy for AMAN. How HBO acts as a M1 to M2 stabilizer, which in this case does not occur in IL-1*β*, requires further research.

## Figures and Tables

**Table 1:** Descriptive data of motoric scale of the rabbit model.

Variable	Group	n	Median	Interquartile	Percentile 25	Percentile 75	*p*
Motoric	C	7	5,00	0,00	5,00	5,00	0.006
	A	7	2,00	2,00	0,00	2,00	
	AH	7	4,00	1,00	4,00	5,00	

**Table 2:** Descriptive data of body weight of the rabbit model.

Variable	Group	n	Mean	SD	*p*
Weight	C	7	3,08	0,07	0.001
	A	7	2,15	0,08	
	AH	7	2,49	0,12	

**Table 3:** Descriptive data of axon degeneration's score of the rabbit model.

Variable	Group	n	Median	Interquartile	Percentile 25	Percentile 75	*p*
Inflammation's infiltration	C	7	1,00	0,00	1,00	1,00	0.045
	A	7	3,00	1,00	3,00	4,00	
	AH	7	2,00	1,00	2,00	3,00	

**Table 4:** Descriptive data in GSH levels of the rabbit model.

Variable	Group	n	Mean	SD	*p*
GSH activity	C	7	189091,71	30382,20	0.007
	A	7	70382,57	193898,78	
	AH	7	132244,00	7657,61	

**Table 5:** Descriptive data of IL-1*β* expression in the rabbit model.

Variable	Group	n	Mean	SD	*p*
IL-1*β* expression	C	7	3,539	0,579	0.561
	A	7	4,071	0,734	
	AH	7	4,714	0,811	

## Data Availability

The data are available at https://http://www.sciencedirect.com/science/article/abs/pii/S1568997216302178.
